# Expanding the clinical and immunological phenotypes of COPB1 deficiency

**DOI:** 10.3389/fimmu.2026.1752685

**Published:** 2026-01-27

**Authors:** Fayhan Alroqi, Thekra Algholaiqa, Sulaiman Alajaji, Abeer Altuwaijri, Nouf Althubaiti

**Affiliations:** 1 Division of Pediatric Allergy and Immunology, Department of Pediatrics, King Abdullah Specialized Children’s Hospital, King Abdulaziz Medical City, Riyadh, Saudi Arabia; 2 King Abdullah International Medical Research Center, Ministry of National Guard-Health Affairs, Riyadh, Saudi Arabia; 3 College of Medicine, King Saud Bin Abdulaziz University for Health Sciences, Riyadh, Saudi Arabia; 4 Department of Clinical Laboratory Sciences, College of Applied Medical Sciences, King Saud Bin Abdulaziz University for Health Sciences, Riyadh, Saudi Arabia

**Keywords:** combined immunodeficiency, inborn errors of immunity, COPB1, COPI, coatomer

## Abstract

**Purpose:**

*COPB1* encodes the coatomer subunit beta protein, which is essential for brain development and intracellular protein trafficking. Homozygous mutations cause Baralle–Macken syndrome that characterized by global developmental delay, severe intellectual disability, and early-onset cataracts. Although immunodeficiency has been observed in patients with COPB1 deficiency, the immunological phenotype remains incompletely characterized. Here, we comprehensively describe the clinical features and delineate the immunological phenotype associated with *COPB1* mutations.

**Methods:**

We performed detailed clinical and immunological evaluations of three female siblings with COPB1 deficiency. Flow cytometry was used to characterize lymphocyte subsets and to assess cytokine secretion following stimulation. Functional proliferation of peripheral blood mononuclear cells (PBMCs) was assessed using dye labeling, CD3/CD28 activation, and flow cytometric analysis.

**Results:**

Three female siblings with COPB1 deficiency presented with early-onset cataracts, global developmental delay, hypotonia, and progressive spasticity leading to quadriplegia. All patients experienced recurrent infections beginning in early childhood. Immunological evaluation revealed neutropenia, T cell lymphopenia, profound reduction in switched and unswitched memory B cells, and absent specific antibody responses. All the three patients were initiated on immunoglobulin replacement therapy and antimicrobial prophylaxis.

**Conclusion:**

Our findings expand the clinical and immunological spectrum of COPB1 deficiency, demonstrating combined immunodeficiency with neutropenia, lymphopenia and impaired specific antibody responses. These results support the classification of COPB1 deficiency as a combined immunodeficiency with syndromic features under the IUIS classification system and emphasize the importance of comprehensive immunological evaluation and early immunoglobulin replacement therapy in patients with *COPB1* mutations.

## Introduction

The advancement of genetic diagnostics has significantly enriched our understanding of immunological disorders over the past decades. According to the most recent report from the International Union of Immunological Societies (IUIS), 559 Inborn Errors of Immunity (IEI) have been identified (1). The prevalence of IEI varies across geographical regions. In areas with high rates of consanguinity, such as the Middle East and North Africa (MENA), the prevalence of autosomal recessive disorders is markedly increased. Approximately 33.1% of IEI-associated genes were first described in patients from the MENA region (2, 3).

IEI are broadly categorized into: immunodeficiencies affecting cellular and humoral immunity, combined immunodeficiency with associated or syndromic features, predominantly antibody deficiencies, diseases of immune dysregulation, congenital defects of phagocytes, defects in intrinsic and innate immunity, autoinflammatory disorders, complement deficiencies, bone marrow failure disorders, and phenocopies of inborn errors of immunity (1). The category of combined immunodeficiencies with associated or syndromic features includes many genetic disorders with neurological involvement, such as purine nucleoside phosphorylase (PNP) deficiency, Wiedemann-Steiner syndrome, Vici syndrome, and Jacobsen syndrome. A high index of suspicion is crucial to recognize immunological abnormalities in patients who present with neurological manifestations and recurrent infections.

Recent reports have described a novel mutation in *COPB1* (OMIM #619255) associated with recurrent infections and neurological features (4). *COPB1* encodes the coatomer subunit beta protein, which plays a critical role in brain development and intracellular protein trafficking. Homozygous mutations in *COPB1* cause Baralle-Macken syndrome, characterized by global developmental delay, severe intellectual disability, and early-onset cataracts (4). Additional clinical features may include metabolic abnormalities, dysmorphic facial features, microcephaly, spasticity, and immunodeficiency. Affected patients typically present in infancy with hypotonia and global developmental delay, with motor impairment ranging from an unsteady gait to complete inability to walk.

To date, this syndrome has been reported in six females and two males from three unrelated consanguineous families of diverse ethnic origins, including Roma (Polish), Saudi, and Pakistani (4, 5). Four of these patients were diagnosed with combined immunodeficiency, presenting with neutropenia, lymphopenia, and impaired antibody responses. They were managed with regular immunoglobulin replacement therapy and prophylaxis against Pneumocystis jirovecii pneumonia (PJP).

Herein, we expand the clinical and immunological phenotype of patients with COPB1 deficiency through comprehensive evaluation of three affected siblings. We propose that COPB1 deficiency should be considered in future IUIS classifications under the category of combined immunodeficiency with associated or syndromic features and neurological manifestations.

## Materials and methods

### Patient recruitment and DNA extraction

Peripheral blood samples were collected in EDTA tubes, and genomic DNA was isolated from whole blood using the QIAsymphony^®^ platform with the DSP DNA Midi Kit (QIAGEN, Hilden, Germany), following the manufacturer’s instructions. Written informed consent was obtained from all adult participants and from parents or legal guardians of minors, including consent for the publication of potentially identifiable data. DNA yield and purity were assessed before proceeding to downstream analyses.

### Whole-genome library preparation and sequencing

Genomic DNA was enzymatically fragmented, and sequencing adapters were attached by PCR to generate Illumina-compatible libraries. Paired-end whole- genome sequencing (WGS) was performed on an Illumina platform to achieve an average depth of approximately 30× across the nuclear genome. Reads were aligned to the human reference genome GRCh37/hg19, and mitochondrial reads were aligned to the revised Cambridge Reference Sequence (rCRS; NC_012920). Variant calling, annotation, and primary filtering were carried out using an integrated in-house/provider bioinformatics pipeline.

### Bioinformatics

Single nucleotide variants (SNVs) and small insertions/deletions (indels) were called across the genome, and copy-number variants (CNVs) were assessed using a DRAGEN-based workflow. Variants were filtered against population frequency data (gnomAD), focusing on those with a minor allele frequency (MAF) <1% in the overall and relevant sub-populations, with rarer alleles prioritized according to the suspected inheritance pattern. Clinical variant databases (HGMD^®^, ClinVar, CentoMD^®^) were queried for previously reported disease-associated variants.

Mitochondrial variants with heteroplasmy levels ≥15% were considered for interpretation. All variants were classified according to ACMG/AMP guidelines (pathogenic, likely pathogenic, variant of uncertain significance, likely benign, benign). Reporting was restricted to variants in genes with established gene– disease associations (OMIM^®^) and with relevance to the patients’ phenotypes. CNVs of uncertain significance were not reported. Variants with low sequencing quality and/or ambiguous zygosity were confirmed by orthogonal methods when clinically indicated.

### Sanger sequencing

Sanger sequencing of the candidate *COPB1* variant was performed in all available family members. The *COPB1* variant (NM_016451.4; c.1651T>G, p.Phe551Val) was amplified by PCR using gene-specific primers designed with Primer3. PCR was performed using standard conditions with an annealing temperature of 60°C. Bidirectional sequencing was carried out by capillary electrophoresis on an ABI 3730xl instrument (Applied Biosystems), and chromatograms were inspected to confirm the presence and segregation of the variant in each tested individual using Mutation Surveyor DNA Variant Analysis Software v5.0 (SoftGenetics, USA).

### Antibodies and flow cytometry

Peripheral blood lymphocyte subsets were analyzed by flow cytometry using fluorochrome-conjugated monoclonal antibodies (CD4 (RPA-T4), CD8 (SK1), CCR7 (G043H7), CD45RA (HI100), CD19 (SJ25C1), CD27 (M-T271), IgD (IA6-2), CD3 (UCHT1), CD56 (MY31), CD16 (CB16), FOXP3 (PCH101), CTLA4/CD152 (14D3), IFN-γ (4S.B3), TNF-α (MAb11), IL-17 (eBlo64DEC17), and appropriate isotype controls). Absolute lymphocyte counts were calculated from complete blood counts and flow cytometric percentages. The methods for extracellular and intracellular staining have been described previously (6).

### Proliferation assay

Fresh peripheral blood mononuclear cells (PBMCs) were isolated and labeled with Cell Proliferation Dye eFluorTM 450 (CPD) (eBioscience) as previously described (7). Briefly, PBMCs were suspended in pre-warmed PBS at a concentration of 3 × 106 cells/ml and labeled with CPD at a concentration of 10 μM (Invitrogen, Oslo, Norway) in a 1:1 cell-to-dye ratio at 37°C for 10 minutes. Labeled cells were collected, washed with fresh medium containing 10% fetal bovine serum (FBS) and antibiotics (penicillin/streptomycin), counted, seeded in 96-well plates (3 × 105 cells/well), and stimulated with Dynabeads^®^ Human T-Activator CD3/CD28- coated beads (Invitrogen Dynal). Unstimulated cells (media only) and unstained cells (negative control) were included as controls. Cell cultures were incubated at 37°C in 5% CO2 for 5 days. After incubation, cells were harvested and analyzed by flow cytometry. Data were further processed using FlowJo software.

## Results

**Figure 1:**
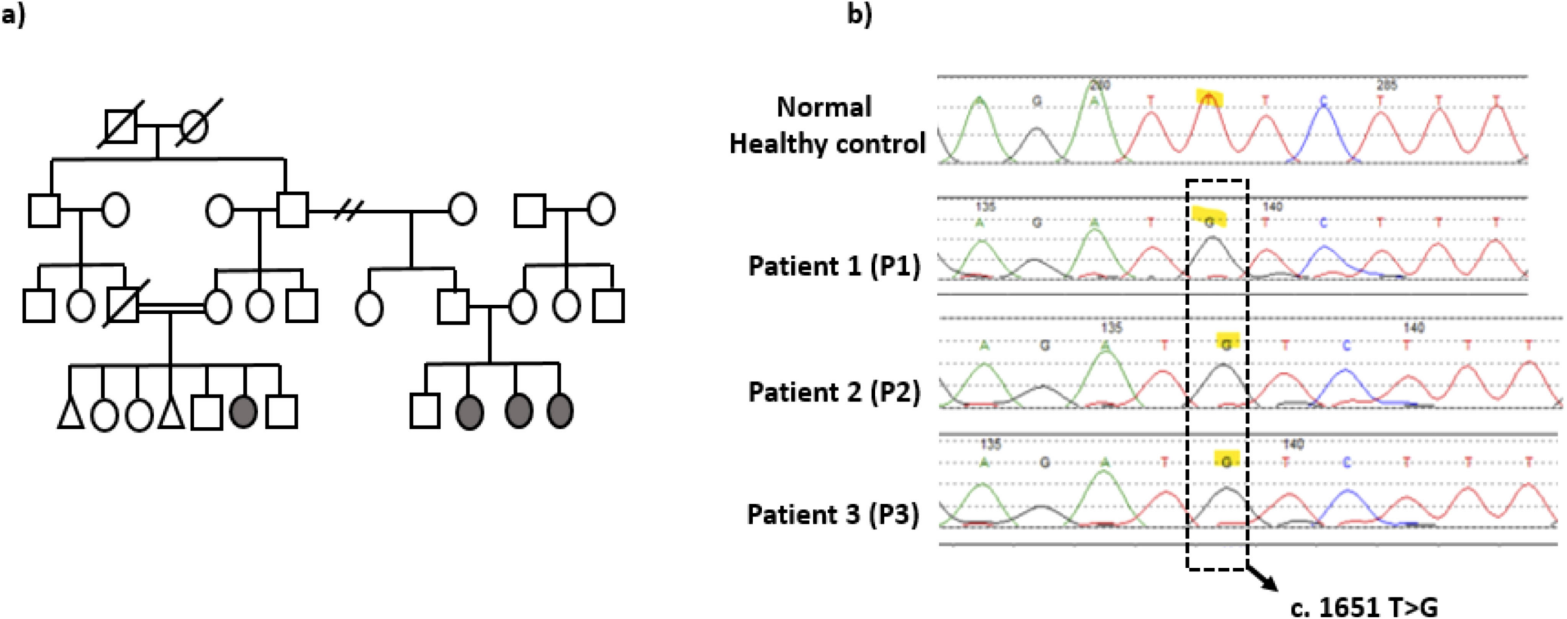
Patient's pedigree and COPB1 gene DNA sequencing chromatogram. **(a)** Pedigree of the family. **(b)** COPB1 gene DNA sequencing chromatogram demonstrated the detection of homozygous mutation c.1651 T>G, missense variant in exon 14 in the patient in compared to healthy control with wild nucleotide sequence.

We conducted a comprehensive clinical and immunological evaluation of three female siblings with COPB1 deficiency (aged 24, 20, and 17 years) born to consanguineous Saudi parents (**Figure 1a**). All siblings presented with early-onset cataracts, global developmental delay, and hypotonia. Over time, they developed spasticity and quadriplegia, becoming wheelchair-bound, as documented in a previous report (4). Patient 1 (P1) experienced recurrent infections starting at 2 years of age. Her initial immunological workup revealed marked neutropenia, lymphopenia, and normal immunoglobulin levels but with absent specific antibody responses to tetanus and polio despite complete vaccinations. A diagnosis of combined immunodeficiency was established, and the patient was initiated on immunoglobulin replacement therapy and Pneumocystis jirovecii pneumonia (PJP) prophylaxis. Patients 2 and 3 (P2, P3) were screened for primary immunodeficiency in early infancy and were found to have similar immunological abnormalities. Both patients were started on immunoglobulin replacement therapy and PJP prophylaxis. Since early childhood, all three siblings have required frequent hospital admissions, primarily due to recurrent viral and cutaneous fungal infections, which were treated with topical and systemic antimicrobial therapies. All three patients have had intermittent positive titers for Cytomegalovirus (CMV) and Epstein-Barr Virus (EBV) viremia without evidence of systemic disease. P3 received antiviral therapy on multiple occasions for high CMV titers and was subsequently started on antiviral prophylaxis.

Given the profound immunological findings, whole-genome sequencing (WGS) was repeated recently for the three siblings to exclude other monogenic variants associated with inborn errors of immunity (IEI) that could explain the phenotype. No additional relevant variants were identified apart from the homozygous *COPB1* missense variant in exon 14 (**Figure 1b**). The pathogenicity of this variant was documented in a previous report, demonstrating that the mutation leads to accumulation of mutant protein throughout the Golgi, whereas wild-type protein is specifically localized to the edges of Golgi stacks due to defective Golgi-to-endoplasmic reticulum (ER) recycling (4).

**Figure 2:**
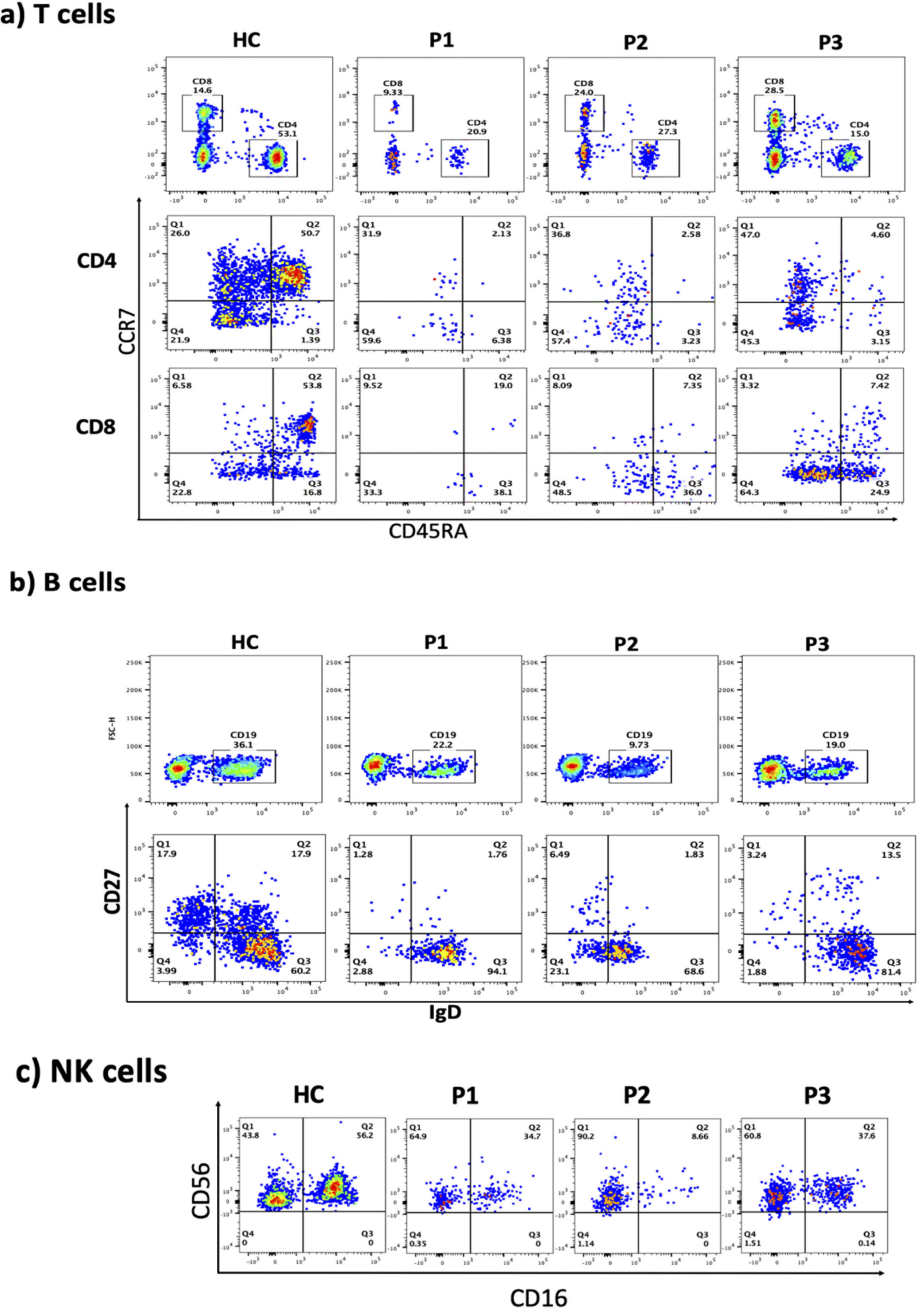
Flow cytometric analyses of: **(a)** CCR7 and CD45RA on CD4 and CD8 T cells, **(b)** CD27 and IgD expressions on CD19 cells, **(c)** CD56 and CD16 on CD3- cells, for healthy control (HC), patient 1, 2, and 3 (P1, P2, P3).

**Figure 3:**
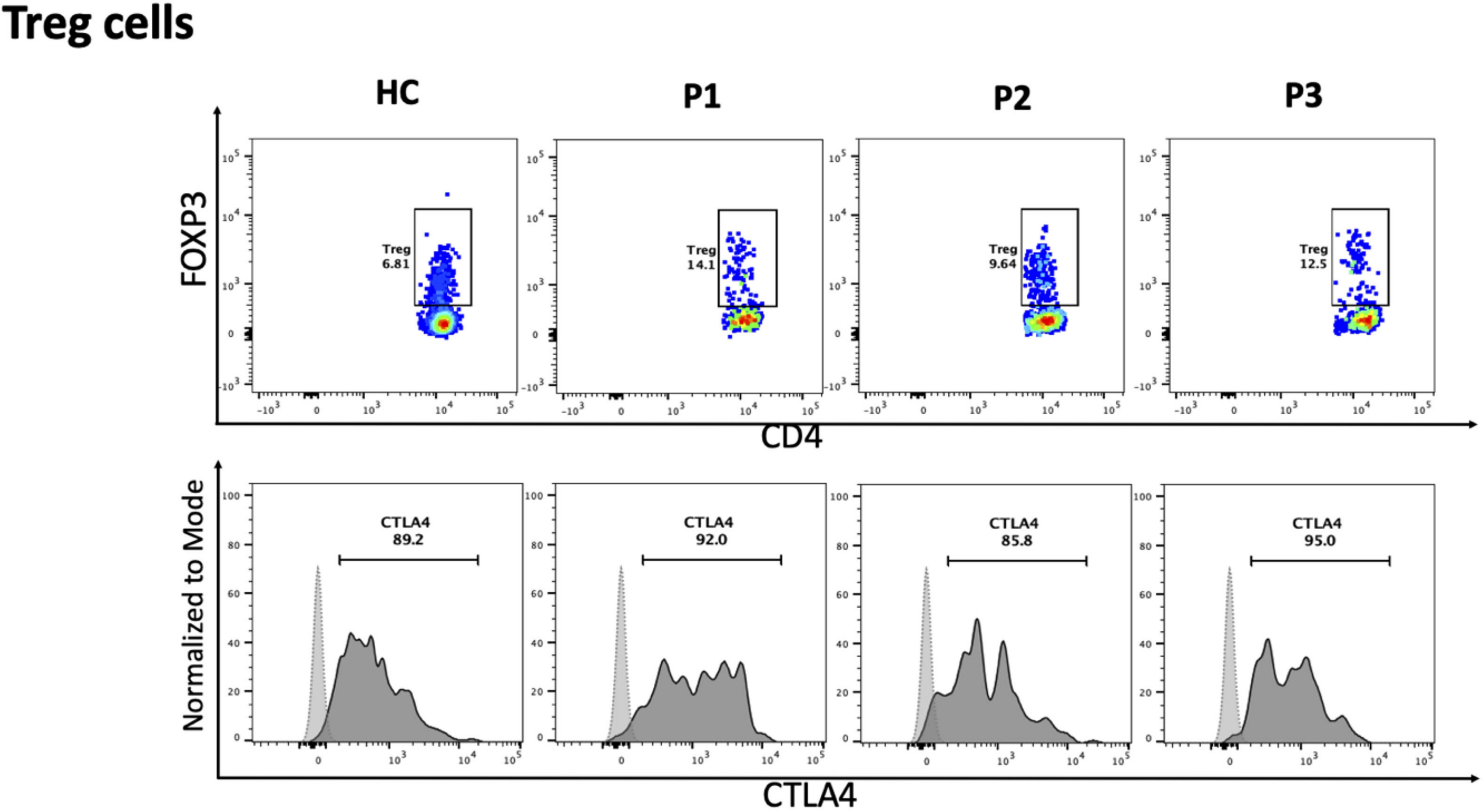
Flow cytometric analyses of FOXP3 on CD4 T cells for Treg, and CTLA4 on Treg, for healthy control (HC), patient 1, 2, and 3 (P1, P2, P3). Fluorescence minus one (FMO) for CTLA4 is presented as light gray dashed line, and expression of CTLA4 is presented as solid line black histogram.

**Figure 4:**
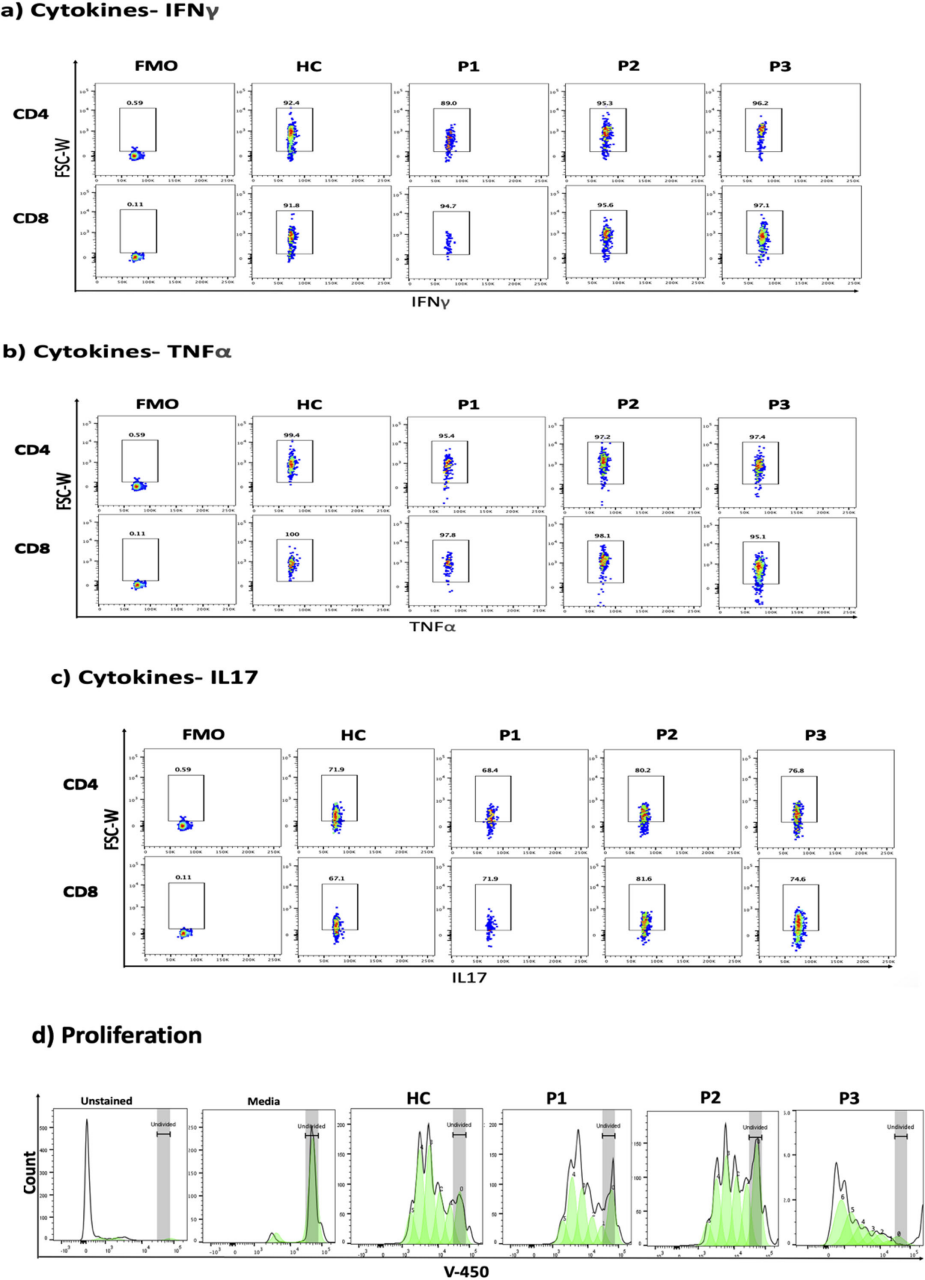
Intracellular expression of **(a)** Interferon-gamma (IFN- γ), **(b)** tumor necrosis factor alpha (TNF-α), **(c)** interleukin-17 (IL17) and **(d)** proliferation analysis. Flow cytometry gating strategy to identify CD4 and CD8 positive subset from lymphocyte and representative of flow cytometry intracellular expression of IFN-γ, TNF-α, and IL17 for healthy control (HC), patient 1, 2, and 3 (P1, P2, P3). Fluorescence minus one (FMO) was used as negative control. **(d)** Proliferation analysis of PBMC after stained with v-450 dye and stimulated with CD3CD28 beads for healthy control (HC), patient 1, 2, and 3 (P1, P2, P3). Unstained PBMC (Unstained), and PBMC stained but unstimulated (Media) were used as control.

The immunological phenotypes of the three siblings are summarized in **Table 1**. Flow cytometric analysis of peripheral blood T lymphocytes demonstrated a marked reduction in naive CD4 and CD8 T cells (CCR7+ CD45RA+) with a relative increase in central and effector memory subsets (**Figure 2a**). All patients exhibited increased frequencies of naive B cells (IgD+CD27low) with a significant decline in both switched and unswitched memory B cells (**Figure 2b**). Evaluation of natural killer (NK) cells showed a marked increase in the CD56+CD16- population (**Figure 2c**). The frequency of regulatory T cells (Tregs) was normal, with normal FOXP3 and CTLA4 expression (**Figure 3**). To further characterize the immunological phenotype, we evaluated intracellular cytokine production and immune cell proliferation upon stimulation. All patients demonstrated normal IFN-γ, TNF-α, and IL-17 secretion after stimulation (**Figures 4a-c**). PBMCs isolated from the three patients displayed normal proliferative responses (**Figure 4d**).

**Table 1:** Lymphocyte subsets immunophenotyping by flow cytometry and immunoglobulin levels.

Parameter	Normal range	Patient 1	Patient 2	Patient 3
Lymphocyte population, cells/mcL				
CD3+	1070 - 2166	44	141	222
CD3+CD4+	559 - 1179	29	72	63
CD3+CD8+	337 - 893	14	60	150
CD19+	100 - 360	33	43	94
CD3-CD16+CD56+	84 - 440	19	45	79
CD19+HLADR+	100%	100%	100%	100%
Immunoglobulins (Ig), g/L – Prior to IVIG				
IgG	6.0 - 16.0	9.94	9.77	8.50
IgM	0.50 - 1.90	0.592	0.502	0.46
IgA	0.80 - 2.80	2.460	1.710	2.58
Vaccine titers				
Tetanus toxoid Ab	> 0.15	0.0	0.07	0.04
Polio antibody titer	≥ 1:8	0.0	0.0	0.0

**Figure 5:**
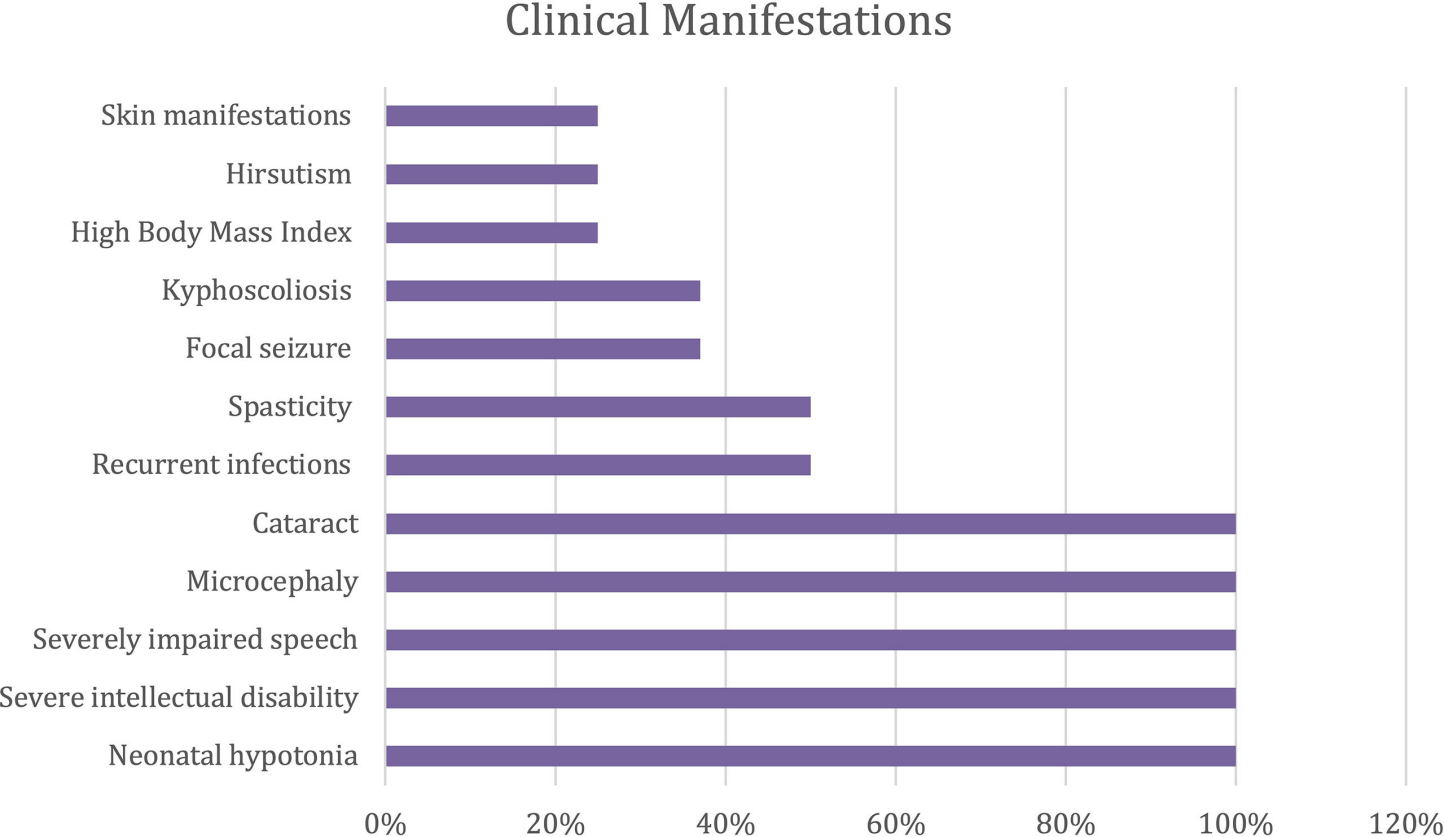
Clinical manifestations in COPB1 deficient patients.

To date, eight patients with COPB1 deficiency have been reported. Previous studies have shown that this disorder encompasses a spectrum of clinical manifestations. Neurological features (neonatal hypotonia, severe intellectual disability, and severely impaired speech), microcephaly, and cataracts are present in all reported patients. A subset of patients developed spasticity (50%) and focal seizures (37%). Kyphoscoliosis was observed in three patients, while hirsutism and skin manifestations, including axillary acanthosis, were present in two patients. Recurrent infections due to impaired immune function were reported in 50% of COPB1- deficient patients (**Figure 5**).

## Discussion

Only eight patients with COPB1 deficiency have been reported in the literature. Although patients share common clinical features of impaired neurological function, microcephaly, and cataracts, profound effects on the immune system have been observed in 50% of cases. Recurrent infections began at an early age, requiring management with immunoglobulin replacement therapy and antimicrobial prophylaxis. Immunological evaluation was consistent with a diagnosis of combined immunodeficiency, with all of our affected patients exhibiting profound lymphopenia, neutropenia, and poor specific antibody responses.

Further immunological phenotyping revealed a marked decrease in naive CD4+ and CD8+ T cells with a significant increase in naive B cells and reduction in memory B cell subsets. We sought to determine whether the combined immunodeficiency phenotype extends beyond quantitative lymphocyte abnormalities and poor specific antibody responses to include functional T-cell defects. We performed comprehensive T-cell functional assessments that revealed normal proliferative responses and cytokines production. Additionally, intact vesicular transport machinery is essential for the expression of multiple molecules including CTLA-4 which is fundamental for the regulatory T cell function. Our results demonstrated normal T regulatory cell frequency and CTLA-4 expression.

Patients with neurological deficits and history of recurrent or severe infections require baseline immunological evaluation to identify possible inborn errors of immunity. Early diagnosis of immunodeficiency and timely initiation of immunoglobulin replacement therapy reduce the risk of infections and help to prevent the development of bronchiectasis (8). Although neurological manifestations may not be reversible, supportive therapy with immunoglobulin replacement and appropriate antimicrobial prophylaxis can significantly improve quality of life and reduce morbidity (9, 10). Our comprehensive evaluation of the three siblings with COPB1 deficiency expands the clinical and immunological phenotype of this rare disorder. The consistent finding of combined immunodeficiency in 50% of reported cases suggests that immunological evaluation should be considered a standard component of clinical assessment in patients with confirmed or suspected COPB1 deficiency.

The association between combined immunodeficiency and *COPB1* mutations has not been extensively characterized in the literature. COPB1 is essential for intracellular protein trafficking, mediating the recycling of proteins from the Golgi apparatus back to the ER (11–14). Defective COPB1 results in ER stress and activation of the unfolded protein response (15). Cells that rely heavily on protein secretion and trafficking are especially vulnerable to ER stress, which may explain the particular susceptibility of immune cells to COPB1 deficiency. Another protein essential for intracellular protein trafficking is the COPI coat complex subunit gamma 1 (COPG1). Mutations in *COPG1* have been reported as a cause of combined immunodeficiency (16, 17). Patients with COPG1 deficiency present with recurrent pulmonary infections, failure to thrive, and susceptibility to persistent EBV and CMV viremia (16). Immunological characteristics include severe CD4+ lymphopenia, poor specific antibody responses, and impaired T-cell proliferation (16). Both COPG1 and COPB1 are core subunits of the COPI coatomer complex and are integral for intracellular trafficking (14). We hypothesize that defects in the *COPB1* gene impair immune cell function through a similar mechanism, specifically through disruption of protein trafficking required for lymphocyte development and antibody production.

This report documents a rare combined immunodeficiency disorder with neurological manifestations and we recommend to include COPB1 deficiency in future IUIS classifications under the category of combined immunodeficiency with associated or syndromic features and neurological manifestations. Some limitations of our study should be acknowledged. First, our cohort consists of three siblings with the same *COPB1* mutation, which limits our ability to assess genotype-phenotype correlations. Larger multicenter studies incorporating patients with different *COPB1* mutations will be important for understanding the full spectrum of immunological manifestations. Second, we did not perform direct experimental assessment of ER stress in patients with COPB1 deficiency. While the known role of COPI in ER-Golgi trafficking suggests that ER stress may contribute to the pathophysiology, this hypothesis requires validation through direct assessment of ER stress markers in patient lymphocytes and see the impact of ER stress on both T and B cells differentiation.

## Data Availability

The original contributions presented in the study are included in the article, further inquiries can be directed to the corresponding author/s.
